# Development of type I/II oligodendrocytes regulated by teneurin-4 in the murine spinal cord

**DOI:** 10.1038/s41598-020-65485-0

**Published:** 2020-05-25

**Authors:** Chikako Hayashi, Nobuharu Suzuki, Riko Takahashi, Chihiro Akazawa

**Affiliations:** 1 Department of Molecular and Cellular Biology, Graduate School of Medical and Dental Sciences, Tokyo Medical and Dental University (TMDU), Tokyo, Japan; 2 Department of Biochemistry and Biophysics, Graduate School of Health Care Sciences, TMDU, Tokyo, Japan; 3 Department of Biochemistry and Biophysics, Graduate School of Medical and Dental Sciences, TMDU, Tokyo, Japan

**Keywords:** Oligodendrocyte, Gliogenesis

## Abstract

In the spinal cord, the axonal tracts with various caliber sizes are myelinated by oligodendrocytes and function as high-velocity ways for motor and sensory nerve signals. In some neurological disorders, such as multiple sclerosis, demyelination of small caliber axons is observed in the spinal cord. While type I/II oligodendrocytes among the four types are known to myelinate small diameter axons, their characteristics including identification of regulating molecules have not been understood yet. Here, we first found that in the wild-type mouse spinal cord, type I/II oligodendrocytes, positive for carbonic anhydrase II (CAII), were located in the corticospinal tract, fasciculus gracilis, and the inside part of ventral funiculus, in which small diameter axons existed. The type I/II oligodendrocytes started to appear between postnatal day (P) 7 and 11. We further analyzed the type I/II oligodendrocytes in the mutant mice, whose small diameter axons were hypomyelinated due to the deficiency of teneurin-4. In the teneurin-4 deficient mice, type I/II oligodendrocytes were significantly reduced, and the onset of the defect was at P11. Our results suggest that CAII-positive type I/II oligodendrocytes myelinate small caliber axons in the spinal cord and teneurin-4 is the responsible molecule for the generation of type I/II oligodendrocytes.

## Introduction

The spinal cord takes on the core roles in our neuronal activity of the central nervous system (CNS). Most of axons are myelinated in the spinal cord white matter (WM), which enables to propagate action potentials rapidly from the brain to the periphery or vice versa. The WM can be divided into 5 different regions: ventral funiculus (VF), lateral funiculus (LF), corticospinal tract (CST), fasciculus cuneatus (FC), and fasciculus gracilis (FG). CST, FC, and FG are collectively called dorsal column (DC)^1^. In the DC, CST and FG consist of the axonal fibers from layer V neurons in the cerebral cortex and the proximal sensory tract from lower limbs, respectively^1,2^. The axonal caliber sizes in the CST and FG are significantly small, compared with the other tract areas. However, these axons are well-myelinated and play a role in our voluntary movements or sensory responses^3^.

Myelin structure that consists of the multi-lamella layer of plasma membranes formed by one of glia cells, oligodendrocyte, acts as the electrical insulator to control the saltatory conduction. A number of studies have demonstrated that myelin formation is also required for neuronal integrity, so that the defects of these structures cause neuronal and mental diseases, such as multiple sclerosis (MS)^4–8^, leukoencephalopathy^9^, and schizophrenia^10–12^. Pathological characteristics of some of these disorders in the spinal cord show that small diameter axons are more vulnerable, compared with large diameter axons^4,7^. For instance, axon staining using post-mortem tissues derived from MS patients displays severer axonal degeneration in the CST and FG of the cervical spinal cord^4^. Furthermore, experimental autoimmune encephalomyelitis (EAE) mice, which are the animal model of MS, often show demyelination in the DC of the spinal cord consisting small diameter axons^13,14^. As above, myelination of small caliber axons is an important phenomenon for functioning of the CNS, and the elucidation of the mechanism should be useful for diagnosis and/or therapy for the related disorders.

In 1928, del Río Hortega identified four types (type I to type IV) of oligodendrocytes with their morphology^15^. Type I/II oligodendrocytes, whose soma is small and round, possess arborized processes and myelinate small diameter axons. Conversely, type III/IV oligodendrocytes form myelin surrounding large diameter axons. They have large and flattened cell bodies with a few processes and form myelin in small number of axons^15–18^. After the discovery by del Río Hortega, Butt and his colleagues identified carbonic anhydrase II (CAII) as a marker for type I/II oligodendrocytes^16,17,19^. They demonstrated that CAII-positive cells in the anterior medullary velum (AMV) extended their complexly arborized processes to the small diameter axons. Regarding CAII staining, the remarkable upregulation of CAII in oligodendrocytes in demyelinated tissues was observed^20,21^. However, the development of CAII-positive type I/II oligodendrocytes in the spinal cord and molecules that regulate their development have not been elucidated yet.

Teneurin-4 (Ten-4), a type II transmembrane glycoprotein, has been identified as a regulator of myelination of small diameter axons^22^. Ten-4 is one of four teneurin family members in vertebrates and is expressed in glia cells including oligodendrocytes^22,23^. Our previous results with the electron microscopy (EM) analysis of 7-week-old mouse spinal cord revealed the defect of myelination in small diameter axons, while these axons were formed^22^. Further, the number of total oligodendrocytes, which were positive with the antibody CC1, was reduced in Ten-4 deficient (−/−) mice^22^. However, an investigation regarding types of oligodendrocytes, particularly type I/II oligodendrocytes, in Ten-4 −/− mice has not been carried out.

In this study, we examined the normal development of CAII-positive type I/II oligodendrocytes in the spinal cord and the association of Ten-4 with type I/II oligodendrocytes by immunohistochemistry and EM analysis. From our results using 7-week-old WT spinal cord, CAII-positive cells were found in the whole spinal cord, but were accumulated in the CST, FG, and the inside part of VF, in which small diameter axons existed. Besides, these cells emerged from postnatal day (P) 7 to 11. In Ten-4 −/− mice, CAII-positive type I/II oligodendrocytes were reduced in the WM. The reduction of these cells was already observed at P11. Our findings indicated that WT type I/II oligodendrocytes myelinate small caliber axons in the CST, FG, and the inside part of VF and their development is regulated by Ten-4. To our knowledge, this is the first report that identified a regulator of specific types (type I/II) of oligodendrocytes.

## Results

### Distribution and development of CAII-positive type I/II oligodendrocytes around small caliber axons in the spinal cord

**Figure 1:**
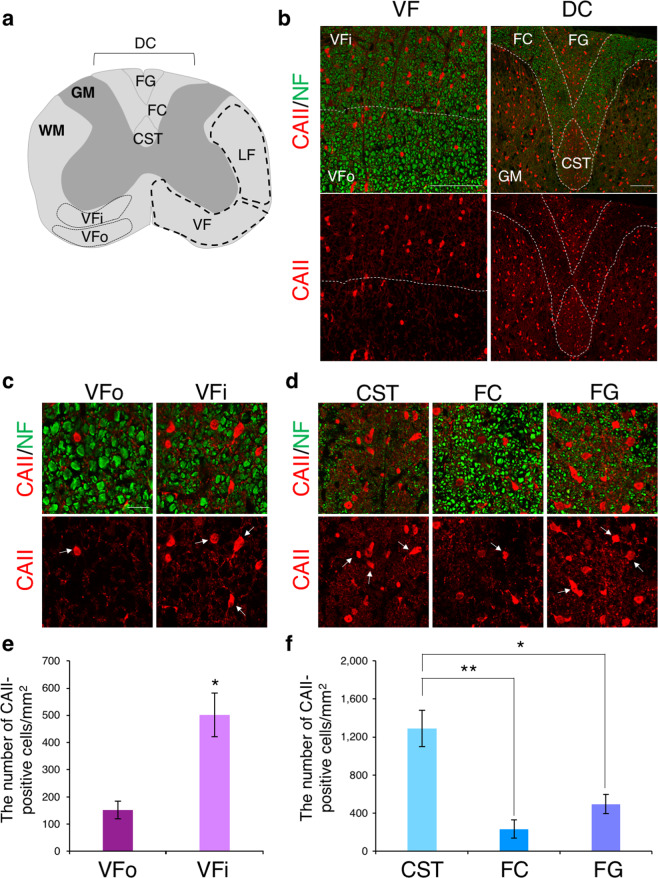
Distribution of CAII-positive oligodendrocytes around small caliber axons. (**a**) Schematic diagram of the rodent spinal cord at the cervical level. The WM in the spinal cord can be divided into 3 regions, ventral funiculus (VF), lateral funiculus (LF), and dorsal column (DC). In this study, the VF is divided into 2 subareas, VFo and VFi, from the respect to the axonal sizes. The DC is also subdivided into 3 areas, corticospinal tract (CST), fasciculus cuneatus (FC), and fasciculus gracilis (FG). (**b**) Immunohistochemical images of carbonic anhydrase II (CAII; red) and neurofilament (NF; green) in the VF and DC of WT mice at 7 weeks. There were various sizes of axons in the white matter (WM) of spinal cord and CAII-positive cells were distributed around small diameter axons. Scale bar: 100 μm. (**c**,**d**) Higher magnification of CAII and NF staining in the VFo and VFi (**c**), and in the CST, FC and FG (**d**). The number of CAII-positive cells was larger in the VFi than in the VFo, and in the CST and FG than in the FC. Arrows: CAII-positive cells. Scale bar: 20 μm. (**e**) Quantitative analysis of the number of CAII-positive cells in the VF. CAII-positive cells were preferably distributed in the VFi. Triplicate experiments were independently performed (n = 3). Error bars represent mean ± s.e.m. The two-tailed Student’s *t*-test was used for the statistical analysis in the experiments with two groups, VFo and VFi. *p < 0.05. (**f**) Quantitative analysis of the number of CAII-positive cells in the DC. More CAII-positive cells were observed in the CST than in the FC. Though the number in the FG was also larger than in the FC, there was not the quantitative difference. Triplicate experiments were independently performed (n = 3). Error bars represent mean ± s.e.m. One-way ANOVA followed by Tukey’s post hoc test for multiple comparisons was used for the statistical analysis. *p < 0.05, **p < 0.01.

To examine the distribution of CAII-positive cells in the spinal cord, we first performed immunostaining in the cervical spinal cord tissues of 7-week-old WT mice using anti-CAII and anti-neurofilament (NF) antibodies to detect type I/II oligodendrocytes and axons, respectively (Fig. 1). In the DC region of the WM, we could confirm that small diameter axons were located in the CST and FG, but larger axons were in the FC, by the immunostaining of NF (Fig. 1a,b,d). The intensity of the NF immunostaining was weak in both the CST and FG, compared with the other areas, because diameters of CST and FG axons were small. With higher exposure of NF immunofluorescence, NF-positive small diameter axons were observed (Supplementary Fig. S1). Also, smaller axons were found in the inside region of the VF, more than in the outside VF region (Fig. 1b,c). Therefore, we subdivided the VF into 2 regions and hereafter designated the inside and outside regions VFi and VFo, respectively (Fig. 1a–c). As a result of CAII staining, CAII-positive type I/II oligodendrocytes were located both in the WM and GM (Fig. 1b). CAII is expressed not only in type I/II oligodendrocytes but also in satellite oligodendrocytes, which adjacently exist with neuronal soma in the GM and unmyelinate axons^20,24^. When we focused on the VF tissues of the WM, the higher number of CAII-positive cells was observed in the VFi than VFo (VFi: 500.8 ± 80.45 cells/mm^2^; VFo: 151.5 ± 31.71 cells/mm^2^) (Fig. 1b,c,e). In the DC, there were more CAII-positive cells in the CST and FG, rather than FC (CST: 1,292 ± 191.5 cells/mm^2^; FG: 495.1 ± 100.5 cells/mm^2^; FC: 233.2 ± 97.87 cells/mm^2^) (Fig. 1b,d,f), while a statistical difference was not obtained in the numbers between FG and FC (Fig. 1f). Also, many puncta of CAII staining, reminiscent of numerous well-branched thin processes that myelinate smaller axons, were observed in the CST and FG (Fig. 1d). Taken together, we found that there were many CAII-positive type I/II oligodendrocytes around the small caliber axons, especially in the VFi, CST, and FG of the WM of the spinal cord.

**Figure 2:**
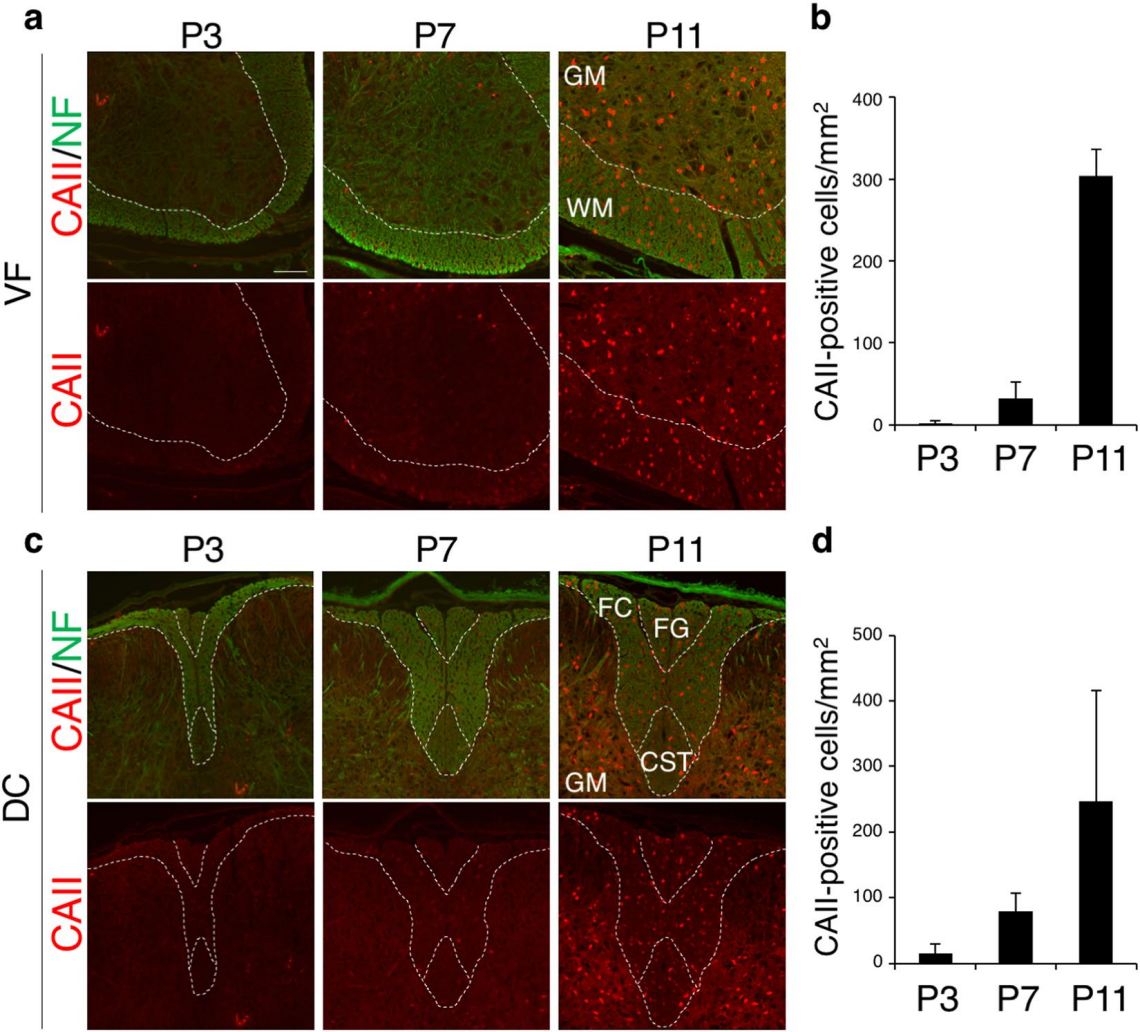
Increased number of CAII-positive cells in the postnatal stages. (**a**,**c**) Immunohistochemical images of CAII (red) and NF (green) in the VF (**a**) and DC (**c**) at P3, P7, and P11 of WT spinal cords. Scale bar: 100 μm. (**b,d**) Quantitative analyses of the number of CAII-positive cells in the VF (**b**) and DC (**d**) during the early postnatal stages. CAII-positive cells were increased at P11. Meanwhile, there were few positive cells at P3. Triplicate or quadruplicate experiments of mice at each postnatal day were independently performed (n = 3 or 4). Error bars represent mean ± s.e.m.

We next examined the development of type I/II oligodendrocytes in the WT spinal cord during the postnatal stages, at P3, P7, and P11 (Fig. 2). Consequently, CAII-positive cells were rarely observed in the whole spinal cord at P3 (VF: 2.399 ± 2.399 cells/mm^2^; DC: 14.73 ± 14.73 cells/mm^2^) (Fig. 2a–d). At P7, CAII-positive cells began to appear at the VF and DC, however the intensity of the CAII expression was low (VF: 32.49 ± 20.17 cells/mm^2^; DC: 79.67 ± 26.56 cells/mm^2^) (Fig. 2a–d). At P11, however, CAII-positive cells were significantly increased in all the regions of the spinal cord (VF: 303.6 ± 32.35 cells/mm^2^; DC: 247.2 ± 169.2 cells/mm^2^) (Fig. 2a–d). These results suggest that CAII-positive type I/II oligodendrocytes appear in the spinal cord at the stage from P7 to P11.

### Reduction of type I/II oligodendrocytes in the spinal cord of Ten-4 −/− mice

In our previous EM analysis, small diameter axons were unmyelinated in the VF/LF of 7-week-old Ten-4 −/− mice^22^. Here, we tested immunostaining of myelin basic protein (MBP) in the cervical spinal cord of Ten-4 −/− mice, and found that the defects of myelin formation of small caliber axon were detected, especially in the CST and FG (Supplementary Fig. S2).

**Figure 3:**
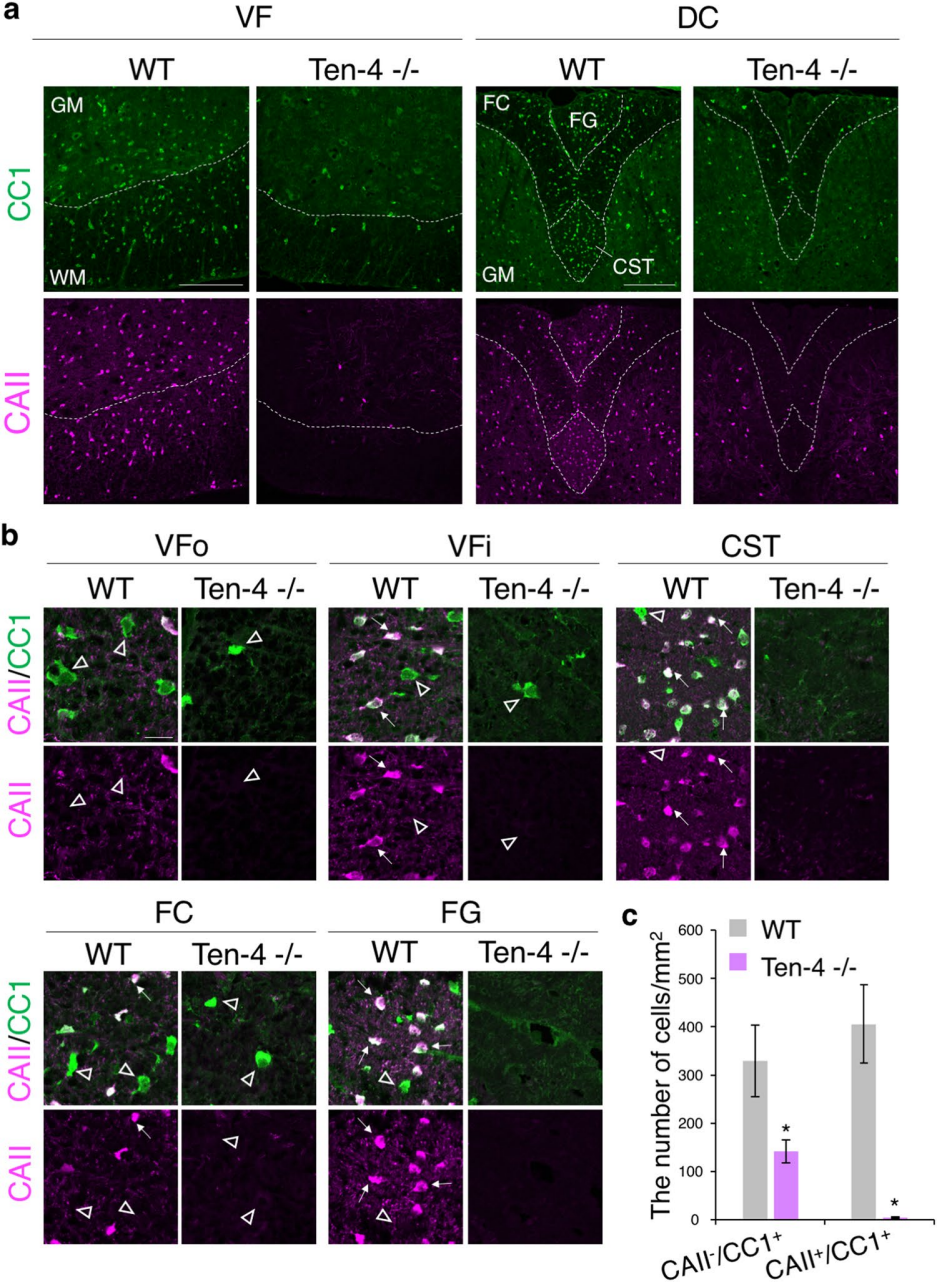
Reduced CAII/CC1-double positive oligodendrocytes in the Ten-4 −/− spinal cord at 7 weeks. (**a**) Immunohistochemical images of CAII (magenta) and CC1 (green) in Ten-4 −/− and WT spinal cords at 7 weeks. CAII/CC1-double positive cells were substantially decreased in the WM in Ten-4 −/− mice. All CAII-positive cells were positive for CC1 and there were 45% of CC1-single positive cells. Scale bar: 200 μm. (**b**) Higher magnification of CAII/CC1-double positive cells in the VFo, VFi, CST, FC, and FG. Double positive cells (white) were reduced in Ten-4 −/− mice. CAII-single positive cells were not observed. Arrows: CAII/CC1-double positive cells. Arrowheads: CC1-single positive cells. Scale bar: 20 μm. (**c**) Quantitative analysis of the number of CC1-single positive and CAII/CC1-double positive cells in the entire WM areas of the spinal cord in WT and Ten-4 −/− mice at 7 weeks. The reduction of CAII/CC1-double positive cells in Ten-4 −/− mice was more prominent than that of CC1-single positive cells. Triplicate experiments were independently performed (n = 3). Error bars represent mean ± s.e.m. The two-tailed Student’s *t*-test was used for the statistical analysis in the experiments with two groups, WT and Ten-4 −/−. *p < 0.05.

We then asked whether CAII-positive type I/II oligodendrocytes were normally developed or not in the Ten-4 −/− spinal cord. Therefore, we performed immunostaining of CAII in mouse tissues at 7 weeks. CAII was expressed in only type I/II oligodendrocytes in the WM and the antibody CC1 was used as a control marker for all types of oligodendrocytes. In the WT spinal cord tissue, about 55% of CC1-positive oligodendrocytes expressed CAII (CC1-single positive cells: 329.0 ± 74.22 cells/mm^2^; CAII/CC1-double positive cells: 405.7 ± 80.95 cells/mm^2^) (Fig. 3a,c). There were no CAII-single positive cells (Fig. 3a,b). Most of CAII/CC1-double positive type I/II oligodendrocytes were located particularly in the VFi, CST, and FG (Fig. 3b: arrows). However, in the Ten-4 −/− spinal cord tissue, CAII/CC1-double positive type I/II oligodendrocytes were dramatically reduced, compared with WT, whereas CC1-single positive oligodendrocytes were also declined but a certain number of them was found in the Ten-4 −/− tissue (CC1-single positive cells: 141.5 ± 23.39 cells/mm^2^; CAII/CC1-double positive cells: 4.208 ± 1.426 cells/mm^2^) (Fig. 3a–c). Though CC1-single positive cells were reduced in a half in Ten-4 −/− mice, CAII/CC1-double positive cells were decreased by 99% compared with WT mice. (Supplementary Fig. S3). The further observation with higher magnification revealed that the larger cell bodies of CC1-single positive cells in both WT and Ten-4 −/− mice were reminiscent to the characteristics of type III/IV oligodendrocytes (Fig. 3b: arrowheads), but not type I/II oligodendrocytes (Fig. 3b: arrows). Hence, Ten-4 is required for either production or maintenance of CAII-positive type I/II oligodendrocytes in the spinal cord.

### Defect of the development of type I/II oligodendrocytes in Ten-4 −/− mice at the postnatal stage

**Figure 4:**
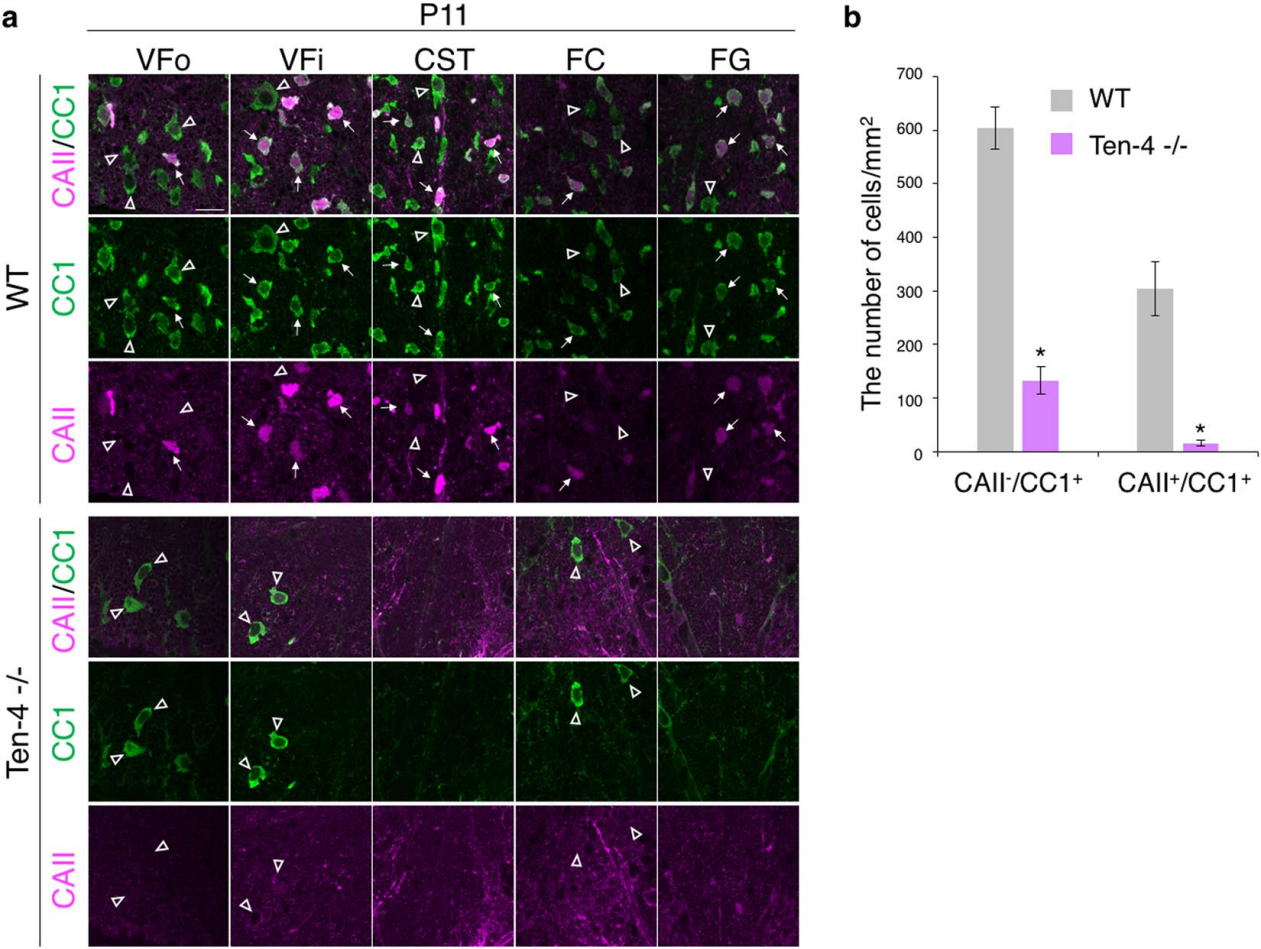
Reduction of CAII/CC1-double positive type I/II oligodendrocytes in Ten-4 −/− mice at the postnatal stage. (**a**) Immunohistochemical images of CAII (magenta) and CC1 (green) at P11 of Ten-4 −/− and WT mice are displayed. CAII/CC1-double positive cells (white) were decreased in the VFi, CST, and FG in Ten-4 −/− mice. CAII-single positive cells were rarely observed both in Ten-4 −/− and WT mice. Arrows: CAII/CC1-double positive cells. Arrowheads: CC1-single positive cells. Scale bar: 20 μm. (**b**) Quantitative analysis of the number of the CC1-single positive and CAII/CC1-double positive cells in the entire WM areas of the spinal cord in WT and Ten-4 −/− mice at P11. The reduction of CAII/CC1-double positive type I/II oligodendrocytes in Ten-4 −/− mice was already observed at P11. Triplicate experiments were independently performed (n = 3). Error bars represent mean ± s.e.m. The two-tailed Student’s *t*-test was used for the statistical analysis in the experiments with two groups, WT and Ten-4 −/−. *p < 0.05.

We further asked whether type I/II oligodendrocytes in the Ten-4 −/− spinal cord were already reduced at the postnatal oligodendrocyte differentiation stage. We used Ten-4 −/− and WT mouse tissues at P11, when the enough number of CAII-positive cells for evaluation was observed in the WT spinal cord (Fig. 2). In consequence, CAII/CC1-double positive type I/II oligodendrocytes were hardly observed in the Ten-4 −/− tissue, while they were seen in WT as expected (WT: 303.6 ± 32.35 cells/mm^2^; Ten-4 −/−: 15.37 ± 3.882 cells/mm^2^) (Fig. 4a: arrows, b). In contrast, CC1-single positive cells were decreased in Ten-4 −/− mice but detected in both Ten-4 −/− and WT mice (WT: 603.9 ± 41.31 cells/mm^2^; Ten-4 −/−: 132.8 ± 21.84 cells/mm^2^) (Fig. 4a: arrowheads, b). CAII/CC1-double positive cells were decreased in Ten-4 −/− mice by 95%, and CC1-single positive cells were 22% out of those in WT mice (Supplementary Fig. S4). There were CAII-single positive cells in neither Ten-4 −/− nor WT tissues. From these data, we concluded that the generation of type I/II oligodendrocyte was inhibited in Ten-4 −/− mice. Interestingly, the CAII staining in punctum- or fiber-like structures, but not cell bodies, was intensified in the Ten-4 −/− tissue (Fig. 4). This implies that Ten-4 deficiency abnormally changes the expression pattern of CAII.

### Onset of the myelination defect of small diameter axons

**Figure 5:**
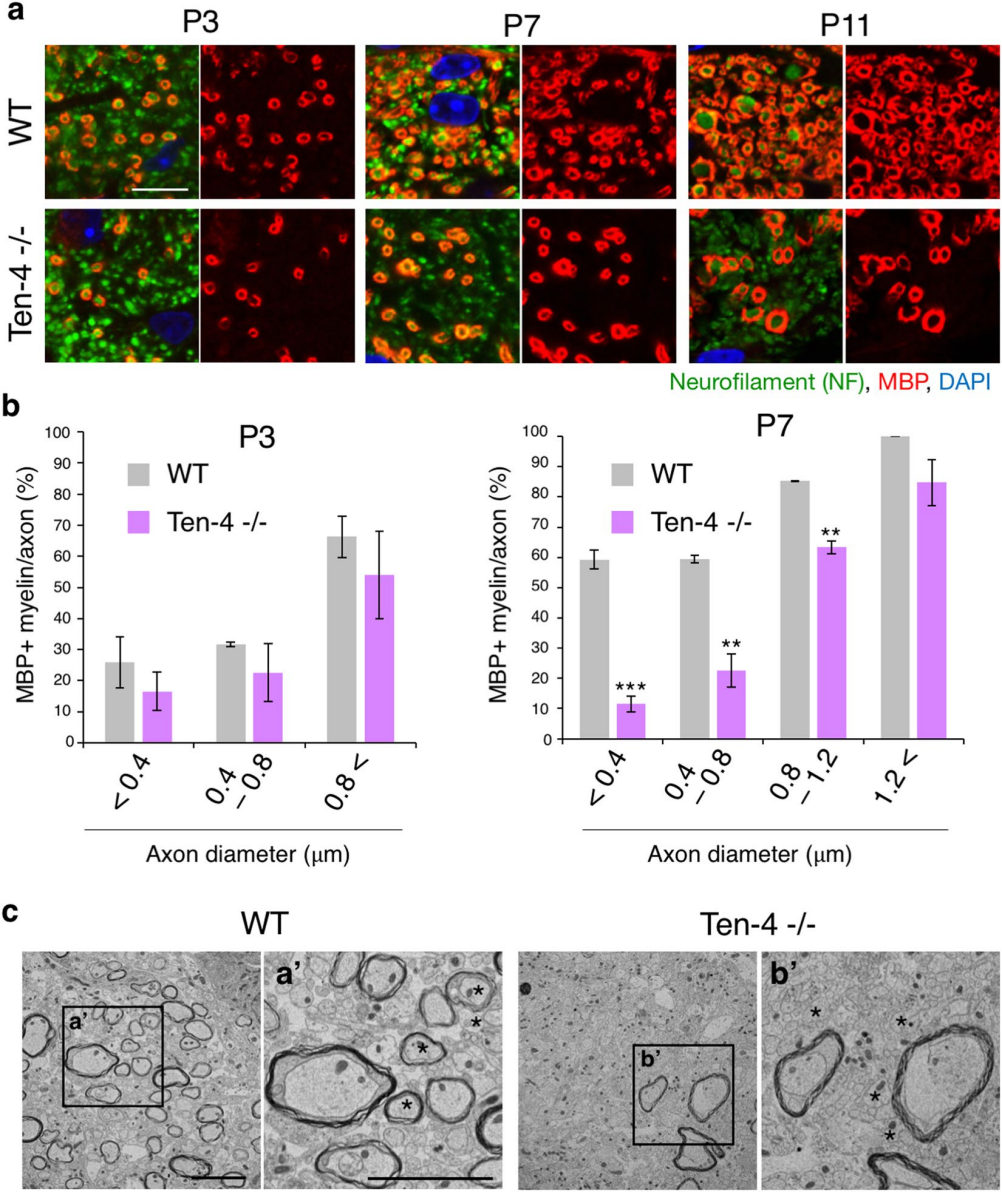
Onset of hypomyelination of small diameter axons in Ten-4 −/− mice. (**a**) Immunohistochemical images of MBP (red) and neurofilament (green) at P3, P7, and P11 of Ten-4 −/− and WT mice. MBP positive myelin around small diameter axons was decreased from P7. The reduction was more obvious at P11. Scale bar: 10 μm. (**b**) Quantitative analysis of myelinated axons with axon diameters in Ten-4 −/− and WT mice. MBP-positive myelin around small diameter axons was diminished from P7 in Ten-4 −/− mice. Triplicate experiments were independently performed (n = 3). Error bars represent mean ± s.e.m. The two-tailed Student’s *t*-test was used for the statistical analysis in the experiments with two groups, WT and Ten-4 −/−. **p < 0.01, ***p < 0.001. (**c**) EM images of myelin and axon structures at P7. Both large and small diameter axons were myelinated in WT mice. Contrary to this, hypomyelination of small axons was observed in Ten-4 −/− mice. (a’,b’) Higher magnification images of indicated boxes. Asterisks: small diameter axons. Scale bar: 5 μm.

Though the hypomyelination of small caliber axons in Ten-4 −/− mice was reported in our previous study^22^, it has not been investigated during the postnatal stage. Therefore, we finally examined whether the myelination of small diameter axons in Ten-4 −/− mice was already interrupted during the postnatal stage or not, due to the reduction of type I/II oligodendrocytes. We assessed the relation of myelination and axonal caliber sizes by immunostaining of MBP and NF at P3, P7, and P11, when myelination began in the WT spinal cord. MBP positive myelinated axons were counted with their diameters. As a result, no differences between WT and Ten-4 −/− tissues were observed in myelination of both small and large diameter axons at P3 (Fig. 5a,b). Larger diameter axons (0.8 μm < ) were preferentially myelinated at this stage. However, at P7, the percentages of myelinated axons were decreased only in smaller diameter axons (<1.2 μm) but not in large diameter axons (1.2 μm < ) in the Ten-4 −/− tissue, compared to WT (Fig. 5a,b). This defect became more obvious at P11 (Fig. 5a). Furthermore, we performed an electron microscopy analysis of the spinal cord tissues at P7. In WT mice, the myelination of both small and large diameter axons was observed (Fig. 5c). By contrast, in Ten-4 −/− mice, myelination of small diameter axons was rarely found at this stage, although large axons were myelinated (Fig. 5c). These results suggest that Ten-4 positively regulates the formation of myelin around the small diameter axons at the beginning of myelination during the early postnatal stage. Together, we conclude that Ten-4 is responsible for the generation of type I/II oligodendrocytes, which myelinate small diameter axons during the initial stage of myelination in the spinal cord.

## Discussion

Our results in this report showed that first, CAII-positive type I/II oligodendrocytes were preferentially located in the small caliber axon areas and initiated to appear from P7 to P11 in the WT spinal cord. Second, we found that type I/II oligodendrocytes were diminished in the Ten-4 −/− spinal cord. Furthermore, the reduction was already started at P11. These results suggest that small caliber axons are myelinated by CAII-positive type I/II oligodendrocytes in the spinal cord during the oligodendrocyte differentiation stage and Ten-4 is required for the generation of type I/II oligodendrocytes for the myelination of small diameter axons.

In our previous study, we found that the differentiation of oligodendrocyte precursor cells (OPCs) into oligodendrocytes and their cell survival were inhibited in Ten-4 −/− mice at P6, although the type of oligodendrocytes was unknown^22^. At the stage, the NF expression and staining as a marker for axon formation was normal in the Ten-4 −/− spinal cord^22^. In the present study, the myelination of the small diameter axons was already inhibited at P7, before high expression of CAII in oligodendrocytes at P11. Around these stages, OPCs interact with axons and receive axonal signals, which trigger their differentiation into oligodendrocytes^25^. In addition, OPCs/oligodendrocytes that fail to adhere and receive the axonal survival factors die through apoptosis^26–28^. Indeed, we recently demonstrated that the extracellular domain of Ten-4 promoted OPCs/oligodendrocytes adhesion and differentiation^29^. From these observations, we speculate that OPCs are unable to interact with smaller axons properly and fail to differentiate into type I/II oligodendrocytes with the expression of CAII in the Ten-4 −/− spinal cord tissue. Additionally, OPCs whose fate is determined into type I/II oligodendrocytes are possibly eliminated through apoptosis before the expression of CAII.

Recently, a couple of groups identified that the lack of expression or function of cell adhesion molecules, such as focal adhesion kinase, β1 integrin, and its ligand laminin α2 chain, caused the defect of myelination in small caliber axons^30,31^. Moreover, cell adhesion is also important for the expression of CAII. In the report by Kida and her colleagues, they demonstrated that CAII was localized in oligodendrocyte processes as well as cell bodies, and CAII-positive varicosities, which were accumulated alongside oligodendrocyte processes, were substantially reduced without the contact with axons^24^. In our study, the reduction of CAII expression including punctuated signals and hypomyelination in small diameter axons were observed in the spinal cord tissue of 7-week-old mice. In addition, our recent report proved that Ten-4 on oligodendrocytes promoted cell-cell adhesion via bindings with teneurins (Ten-1 to Ten-4) on axons, which positively regulated myelin formation^29^. Ten-4 may regulate the cell adhesion to small caliber axons for the differentiation/maturation of CAII-positive type I/II oligodendrocytes.

Hypomyelination and axon degeneration in small caliber axons of the spinal cord are observed in some myelin-related disorders. In Pelizaeus-Merzbacher disease (PMD) patients with the null mutation in the proteolipid protein 1 gene (PLP1), which is a major myelin protein as well as MBP, axon degeneration was observed in the CST and FG^32^. Furthermore, in MS patients, small diameter axons in the CST and FG were degenerated^4,5^. Pathological character of MS in acute phase is that the cycle from demyelination to remyelination is repeated in the various CNS tissues^8^. Interestingly, according to the autopsy samples from acute MS patients, CAII-positive cell number and the expression level of CAII were transiently increased, compared with those from the healthy control samples, though oligodendrocytes positive for the other marker CNPase were not changed. The increased CAII-positive cells may be associated with the activated remyelination of small diameter axons^20^. The other report showed that central pontine myelinolysis (CPM) patients displayed the remarkable upregulation of CAII expression in oligodendrocytes^21^. In this study, we found that the signal of CAII expression in Ten-4 −/− mice at P11 was temporally intensified, in spite of little number of CAII-positive cells. From these evidences, it is possible that Ten-4 may be involved in the pathological conditions of these de- or dys-myelinating diseases through the regulation of CAII-positive type I/II oligodendrocytes. Ten-4 could be a marker in the pathogenesis of these diseases.

Myelin structure of small caliber axons plays important roles for not only body movements but also cognitive functions. Indeed, a MRI analysis revealed that the defect of small caliber axons was observed in the brain of MS patients, whose symptom was cognitive impairments^7^. From the recent interesting studies, chronic oral administration of _D_-aspartate (D-Asp) improved the function of memory, nevertheless the mechanism has not been clarified yet^33^. Also, AMPA receptor agonists promoted the myelination and oligodendrocyte differentiation^34^. D-Asp treatment increased the small caliber axonal activity, so that the activity dependent remyelination selectively occurred^35^. In conclusion of these reports, the promoted activity in the small caliber axons by the AMPA receptor agonists or D-Asp may be useful for the therapy for these myelin-related disorders including cognitive deficiency^35–37^. Though the association of type I/II oligodendrocyte with these mental disorders has not been demonstrated, our present report may shed light on the relation.

In summary, we could identify Ten-4 as a regulator of type I/II oligodendrocytes. To our knowledge, Ten-4 is the first responsible regulator of specific types (type I/II) of oligodendrocytes. Because the neurological and mental functions in the CNS are regulated by various sizes of myelinated axons, the identification of regulating molecules of myelination depending on axonal sizes is necessary. Importantly, Ten-4 has been already identified as the associated gene with neurological and mental diseases, such as essential tremor, bipolar disorder, and schizophrenia^38–40^. Our findings will contribute to the elucidation of the molecular mechanisms for understanding oligodendrocyte biology and may be useful for the development of diagnostic or therapeutic reagents in the related diseases.

## Methods

### Mice

The Ten-4 −/− mouse line (*furue*) that we characterized previously^22^ was kindly provided by Dr. Yoshihiko Yamada from NIDCR, NIH. Littermates at P3, P7, P11, and 7 weeks were used for experiments. All procedures for experimental animals were approved by the Institutional Animal Care and Use Committees of Tokyo Medical and Dental University (Protocol No: 017028C). All methods were conducted in strict accordance with the approved guidelines of the institutional animal care committees.

### Immunohistochemistry

For the preparation of spinal cord tissue sections, P3, P7, and P11 mice were perfused with 5 ml of phosphate buffered saline (PBS) and following fixed with 5 ml of 4% paraformaldehyde (PFA, Wako) in PBS. Fifteen ml of PBS and 15 ml of 4% PFA were used for perfusion of mice at 7 weeks. The spinal cords were dissected out and post-fixed with 4% PFA in PBS. After the post-fixation overnight at 4 °C, the vertebra surrounding the spinal cord tissue at P11 and 7 weeks was removed, spinal cord tissues were soaked into 15% sucrose in PBS, and placed overnight at 4 °C. Then, the tissues were put into 30% sucrose in PBS for 2 days at 4 °C, and were frozenly embedded with O.C.T compound (Sakura Finetek) on dry ice. After that, the spinal cord tissues were sliced into 8 μm sections with cryostat (Leica LCM). The tissue sections were dried for 1 to 2 h, and the sections were fixed with 4% PFA in PBS for 10 min at room temperature and were washed with PBS 3 times (excessive fixation caused a reduction in the reactivity of anti-CAII antibody). To retrieve the antigen, the sections were heated with Target Retrieval Solution, Citrate pH 6.1 (Dako) by microwave. After blocking with Power Block Universal Blocking Reagent (BioGenex Laboratories) for 1 h at room temperature, primary antibodies, rabbit anti-CAII (1:250; Sigma-Aldrich), mouse anti-APC (clone CC1) (1:500; Millipore), rabbit anti-MBP (1:100; Millipore), and rabbit anti-neurofilament (1:500; Sigma-Aldrich), in 1% bovine serum albumin (BSA) in PBS, were incubated overnight at 4 °C. After washing with PBS for 3 times, the sections were labeled with secondary antibodies, rabbit IgG-Alexa 488 (ThermoFisher Scientific), rabbit IgG-Alexa 594 (ThermoFisher Scientific), mouse IgG-Alexa 488 (ThermoFisher Scientific) and mouse IgG-Alexa 594 (ThermoFisher Scientific) for 50 min at room temperature. After 3 times washing with PBS, the samples were mounted with Vectashield containing DAPI (Vector Laboratories). The fluorescent images were taken and analyzed by the fluorescence microscope BZ-X700 (Keyence) or the confocal microscope LSM 700 ZEN (Zeiss). In all immunohistochemical analyses, triplicate or quadruplicate independent experiments gave similar results. Also, the specificity of anti-CAII and other cell-type marker antibodies was validated (Supplementary Fig. S5).

### Electron microscopy analysis

The spinal cords of P7 pups were dissected out and fixed with 2% PFA, 2% glutaraldehyde, and 2.5% sucrose in 0.1 M cacodylate buffer, including 2% osmium tetroxide, for 3 h at 4 °C. After fixation, the tissues were washed with 0.1 M cacodylate buffer 3 times. The following procedures of transmission electron microscopy analysis were performed by Tokai Electron Microscopy Analysis Co., Ltd.

### Statistical analyses

All of the experiments were independently performed at least 3 times. The two-tailed Student’s *t*-test was used for the analyses in the experiments with two groups. One-way ANOVA followed by Tukey’s post hoc test was used for the statistical analysis for multiple comparisons. The statistical significance was defined as *p < 0.05, **p < 0.01, and ***p < 0.001.

## Supplementary information

Supplementary Information.

## Data Availability

All the data are presented in the manuscript as figures.
