# Supplementary material for: Development of type I/II oligodendrocytes regulated by teneurin-4 in the murine spinal cord
Source: Sci Rep. 2020 May 25;10:8611. doi: 10.1038/s41598-020-65485-0 (PMC7248063; doi:10.1038/s41598-020-65485-0)
Supplement: Supplementary file 1 — Supplementary Information. [file 41598_2020_65485_MOESM1_ESM.pdf]

Supplementary Information

Development of type I/II oligodendrocytes regulated by teneurin-4 in the murine spinal cord

Chikako Hayashi[1,2 ¶], Nobuharu Suzuki[1,2 ¶ *], Riko Takahashi[1], and Chihiro Akazawa[2,3*]

[1]Department of Molecular and Cellular Biology, Graduate School of Medical and Dental Sciences, Tokyo Medical and Dental University (TMDU), Tokyo, Japan
[2]Department of Biochemistry and Biophysics, Graduate School of Health Care Sciences, TMDU, Tokyo, Japan
[3]Department of Biochemistry and Biophysics, Graduate School of Medical and Dental Sciences, TMDU, Tokyo, Japan

*To whom correspondence should be addressed: Nobuharu Suzuki, Ph.D., Department of Molecular and Cellular Biology, Graduate School of Medical and Dental Sciences, TMDU, 1-5-45 Yushima, Bldg 3, Bunkyo-ku, Tokyo, Japan, 113-8510, E-mail: nsuzbb@tmd.ac.jp, Tel: +81-3-5803-5364; Fax: +81-3-5803-5364, and Chihiro Akazawa, MD., Ph.D., Department of Biochemistry and Biophysics, Graduate School of Medical and Dental Sciences, TMDU, 1-5-45 Yushima, Bldg 3, Bunkyo-ku, Tokyo, Japan, 113-8510, E-mail: c.akazawa.bb@tmd.ac.jp, Tel: +81-3-5803-5362; Fax: +81-3-5803-5362

¶ These authors equally contributed to this work.

Supplementary Fig. S1: Small caliber axons and CAII-positive type I/II oligodendrocytes in the CST and FG in the spinal cord at 7 weeks.

Supplementary Fig. S2: Hypomyelination in the spinal cord of Ten-4 -/- mice at 7 weeks.

Supplementary Fig. S3: Relative reduction of CAII/CC1-double positive cells in the spinal cord of Ten-4 -/- mice at 7 weeks.

Supplementary Fig. S4: Onset of the relative reduction in the number of CAII/CC1-double positive type I/II oligodendrocytes in the spinal cord of Ten-4 -/- mice at the postnatal stage.

Supplementary Fig. S5: Validation of antibodies specificity.

Figure S1

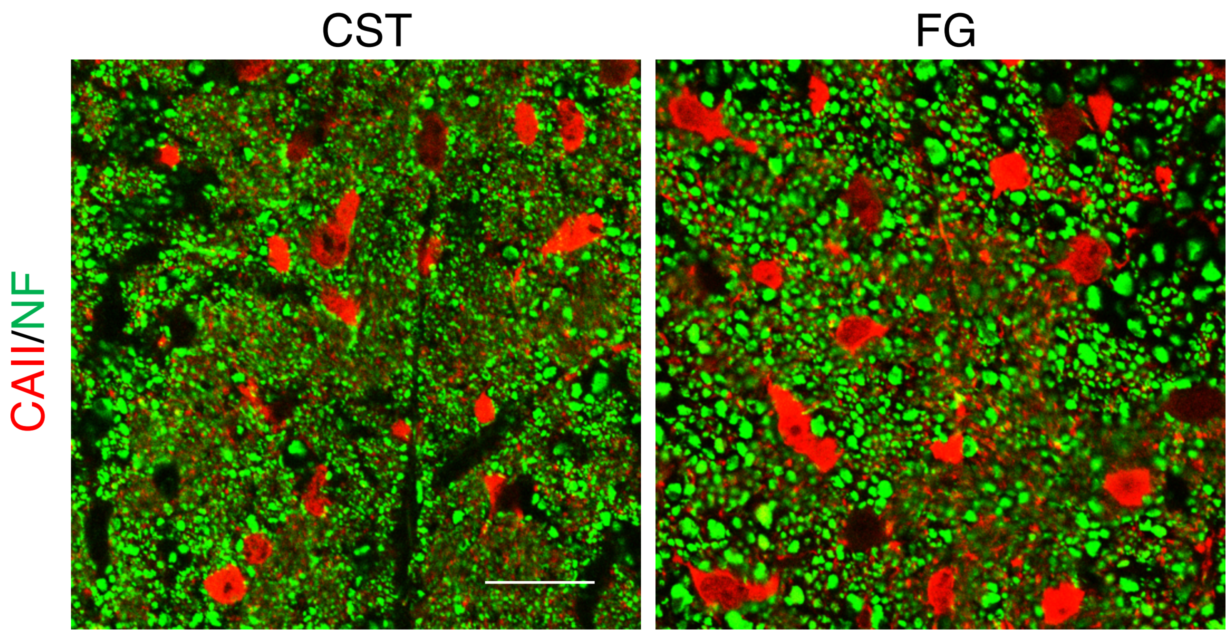

Figure S1. Small caliber axons and CAII-positive type I/II oligodendrocytes in the CST and FG in the spinal cord at 7 weeks. Images with higher exposure of NF immunefluorescence in the CST and FG. CAII-positive cells (red) around NF-positive small caliber axons (green) were observed. Scale bar: 20 µm.

Figure S2

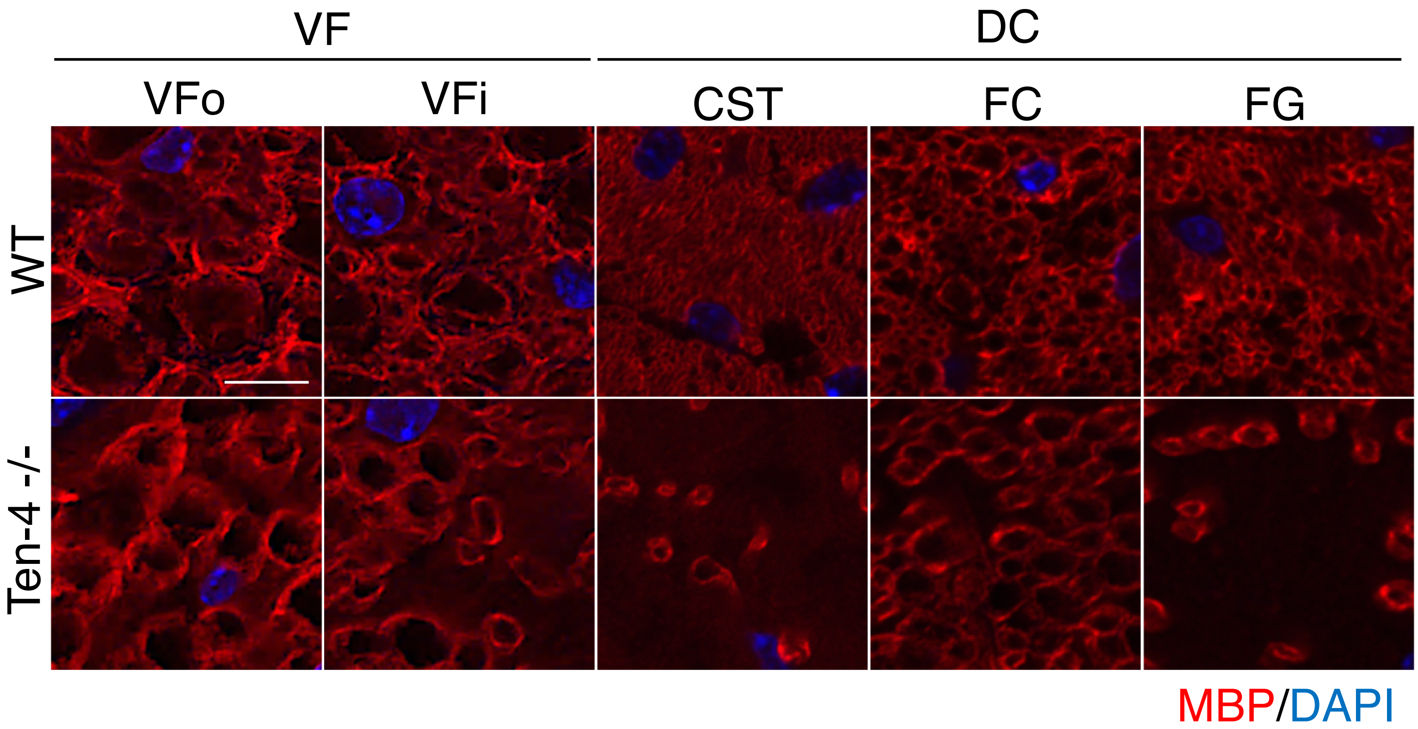

Figure S2. Hypomyelination in the spinal cord of Ten-4 -/- mice at 7 weeks. Higher magnification of MBP immunostaining in the VFo, VFi, CST, FC, and FG. MBP-positive myelin (red) around small caliber axons was decreased, particularly in the CST and FG, which were occupied by small diameter axons. DAPI (blue) was used for nucleus staining. Scale bar: 10 μm.

Figure S3

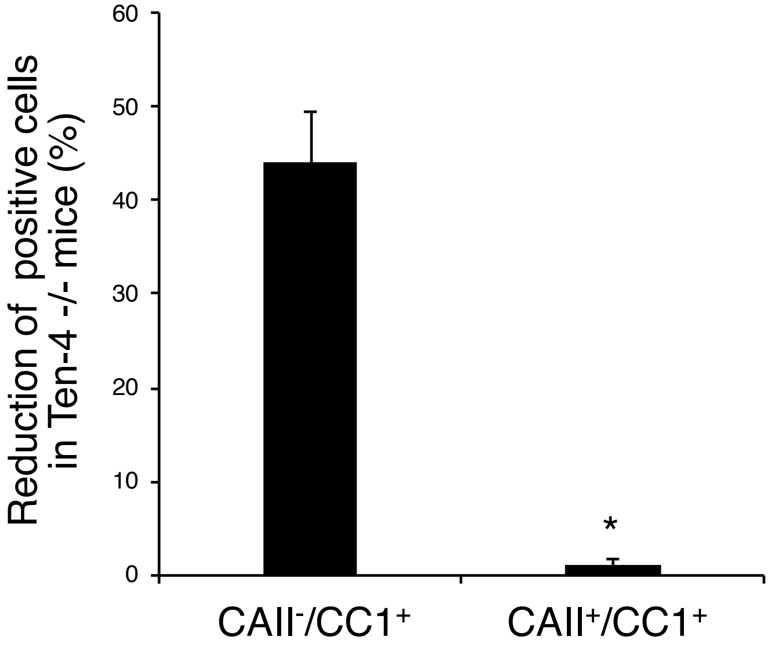

Figure S3. Relative reduction of CAII/CC1-double positive cells in the spinal cord of Ten-4 -/- mice at 7 weeks. Quantitative analysis of CC1-single positive cells and CAII/CC1-double positive cells in the entire WM areas of the spinal cord in WT and Ten-4 -/- mice. The percentages of both CC1-single positive cells and CAII/CC1-double positive cells in the Ten-4 -/- spinal cord out of those cell numbers in WT are indicated. Though both CC1-single positive cells and CAII/CC1-double positive cells were decreased in the Ten-4 -/- tissue, the reduction of CAII/CC1-double positive cells was more prominent. Triplicate experiments were independently performed (n = 3). Error bars represent mean ± s.e.m. The two-tailed Student's *t*-test was used for the statistical analysis in the experiments with two groups. $*p < 0.05$.

Figure S4

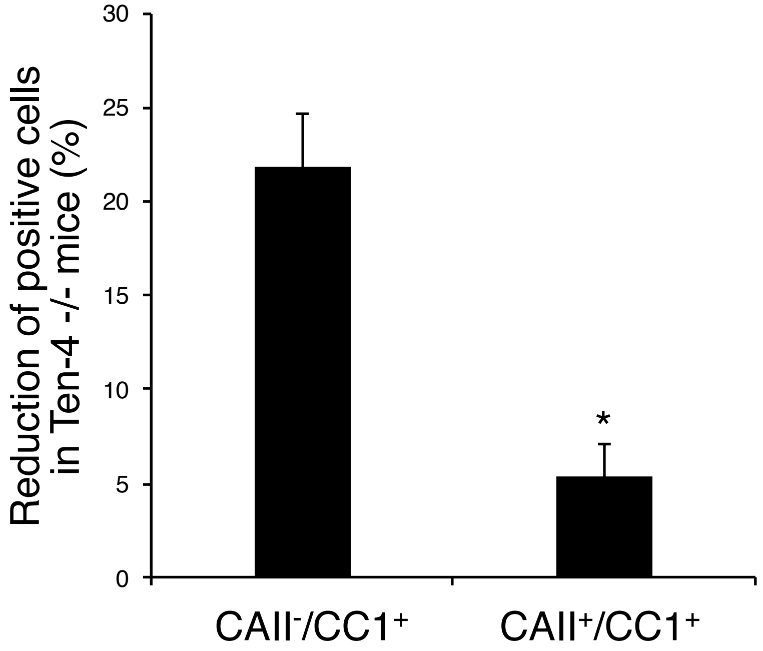

Figure S4. Onset of the relative reduction in the number of CAII/CC1-double positive type I/II oligodendrocytes in the spinal cord of Ten-4 -/- mice at the postnatal stage. Quantitative analysis in of CC1-single positive cells and CAII/CC1-double positive cells in the entire WM areas of the spinal cord in WT and Ten-4 -/- mice at P11. The percentages of both CC1-single positive cells and CAII/CC1-double positive cells in the Ten-4 -/- spinal cord out of those cell numbers in WT are indicated. The significant reduction of CAII/CC1-double positive cells was already observed at P11. Triplicate experiments were independently performed (n = 3). Error bars represent mean ± s.e.m. The two-tailed Student's *t*-test was used for the statistical analysis in the experiments with two groups. $*p < 0.05$.

Figure S5

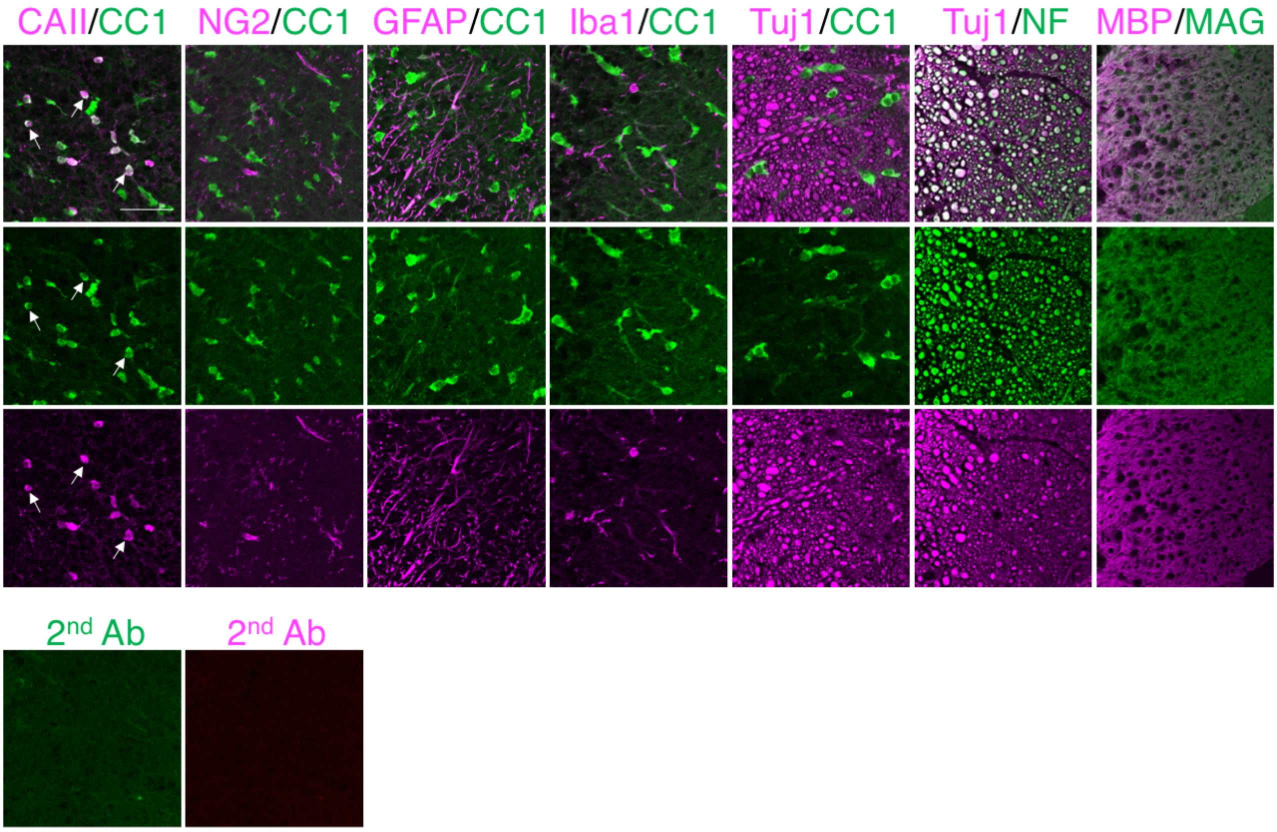

Figure S5. Validation of antibodies specificity. CC1-staining (green) was combined with not only CAII but also other staining of cell-type specific markers (magenta). CC1 for all type of differentiated oligodendrocytes, NG2 for oligodendrocyte precursor cells (OPCs), GFAP for astrocytes, Iba1 for microglia, and Tuj1 for neurons were used as the cell-type specific markers. All CAII-positive cells expressed CC1. CC1-staining was not colocalized with any other cell-type specific markers. Therefore, CAII was expressed specifically on oligodendrocytes. Also, to validate the specificity of NF and MBP antibodies, the staining was combined with Tuj1 and MAG, respectively. NF-positive axons expressed Tuj1, and MBP-positive myelin was positive for MAG. Any signal was undetectable without 1st antibodies (the cell-type specific antibodies described above). Arrows: CAII/CC1-double positive cells. Scale bar: 50 μm.
